# Supplementary material for: Association of plasma and CSF cytochrome P450, soluble epoxide hydrolase, and ethanolamide metabolism with Alzheimer’s disease
Source: Alzheimers Res Ther. 2021 Sep 6;13:149. doi: 10.1186/s13195-021-00893-6 (PMC8422756; doi:10.1186/s13195-021-00893-6)
Supplement: Supplementary file 1 — Additional file 1 : Figure S1. Bile acids biosynthesis pathway. Node colors represent primary (blue) and secondary (dark red) bile acids. Node shape represent conjugated (diamond) and unconjugated (oval) bile acids. Node size represents median concentration in the experimental cohort. Cholesterol (top of the pathway) is converted to primary bile acids along two pathways, neutral and acidic [1]. Further, primary bile acids are secreted to gut, and portion of it being modify by the gut bacteria to secondary bile acids. Primary and secondary bile acids are reabsorbed into the blood stream and reenter the liver, where they are conjugated with amino acids glycine or taurine. Conjugated bile acids are then being secreted back to the gut along with primary bile acids. Gut bacteria can cleave conjugated amino acids off bile acids [2], and freed metabolites are recirculated. Therefore, plasma levels of conjugated bile acids can me a reflection of both liver and gut bacteria activity. Refference: 1.Pandak, W.M. and G. Kakiyama, The acidic pathway of bile acid synthesis: Not just an alternative pathway(). Liver Res, 2019. 3(2): p. 88-98; 2. Ridlon, J.M., et al., Consequences of bile salt biotransformations by intestinal bacteria. Gut Microbes, 2016. 7(1): p. 22-39. [file 13195_2021_893_MOESM1_ESM.pdf]

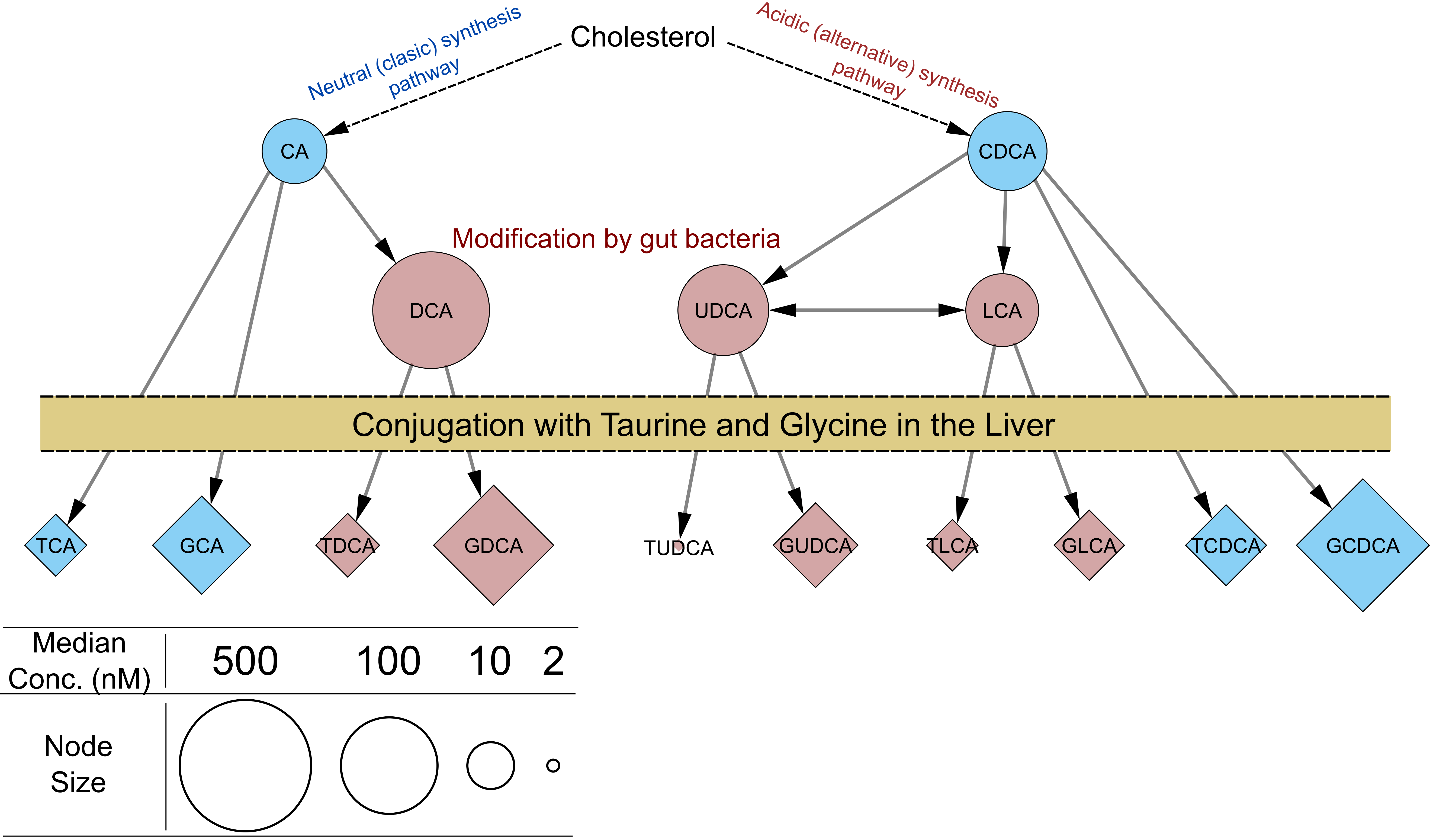

Figure S1. Bile acids biosynthesis pathway. Node colors represent primary (blue) and secondary (dark red) bile acids. Node shape represent conjugated (diamond) and unconjugated (oval) bile acids. Node size represents median concentration in the experimental cohort. Cholesterol (top of the pathway) is converted to primary bile acids along two pathways, neutral and acidic [1]. Further, primary bile acids are secreted to gut, and portion of it being modify by the gut bacteria to secondary bile acids. Primary and secondary bile acids are reabsorbed into the blood stream and reenter the liver, where they are conjugated with amino acids glycine or taurine. Conjugated bile acids are then being secreted back to the gut along with primary bile acids. Gut bacteria can cleave conjugated amino acids off bile acids [2], and freed metabolites are recirculated. Therefore, plasma levels of conjugated bile acids can me a reflection of both liver and gut bacteria activity. Refference: 1.Pandak, W.M. and G. Kakiyama, The acidic pathway of bile acid synthesis: Not just an alternative pathway(). Liver Res, 2019. 3(2): p. 88-98; 2. Ridlon, J.M., et al., Consequences of bile salt biotransformations by intestinal bacteria. Gut Microbes, 2016. 7(1): p. 22-39.
