# Supplementary material for: Association of plasma and CSF cytochrome P450, soluble epoxide hydrolase, and ethanolamide metabolism with Alzheimer’s disease
Source: Alzheimers Res Ther. 2021 Sep 6;13:149. doi: 10.1186/s13195-021-00893-6 (PMC8422756; doi:10.1186/s13195-021-00893-6)
Supplement: Supplementary file 2 — Additional file 2 : Figure S2. Multilinear regression of log(t-Tau/AB42) and the components of AD predictive models, presented in the Figure 4. Analysis performed separately for plasma (upper panel) and CSF (lower panel) AD predictors. Association of individual components are shown in the leverage plots, whereas effect summery contain descriptive statistics for each individual metabolite in the model. [file 13195_2021_893_MOESM2_ESM.pdf]

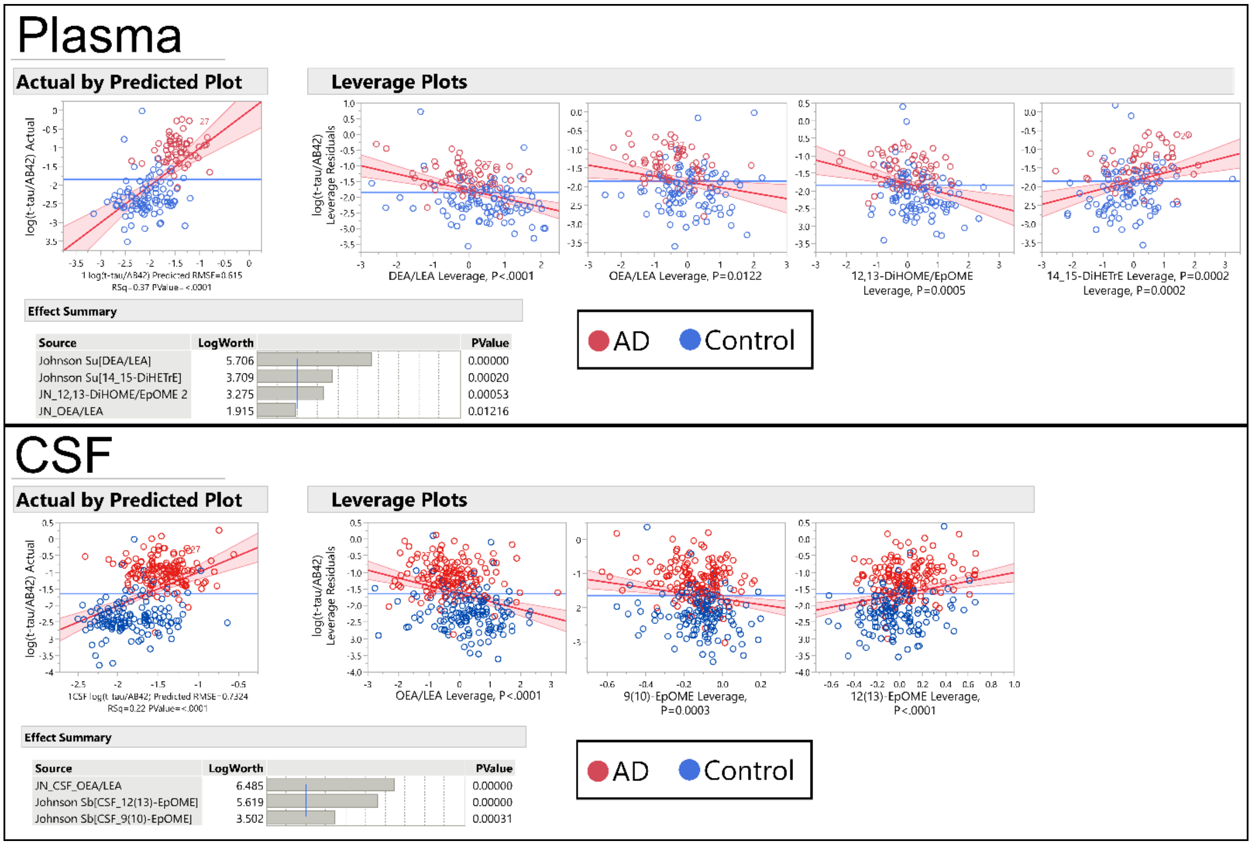

Figure S2. Multilinear regression of log(t-Tau/AB42) and the components of AD predictive models, presented in the figure 4. Analysis performed separately for plasma (upper panel) and CSF (lower panel) AD predictors. Association of individual components are shown in the leverage plots, whereas effect summery contain descriptive statistics for each individual metabolite in the model.
