# Supplementary figures and images for: Association of plasma and CSF cytochrome P450, soluble epoxide hydrolase, and ethanolamide metabolism with Alzheimer’s disease
Source: Alzheimers Res Ther. 2021 Sep 6;13:149. doi: 10.1186/s13195-021-00893-6 (PMC8422756; doi:10.1186/s13195-021-00893-6)

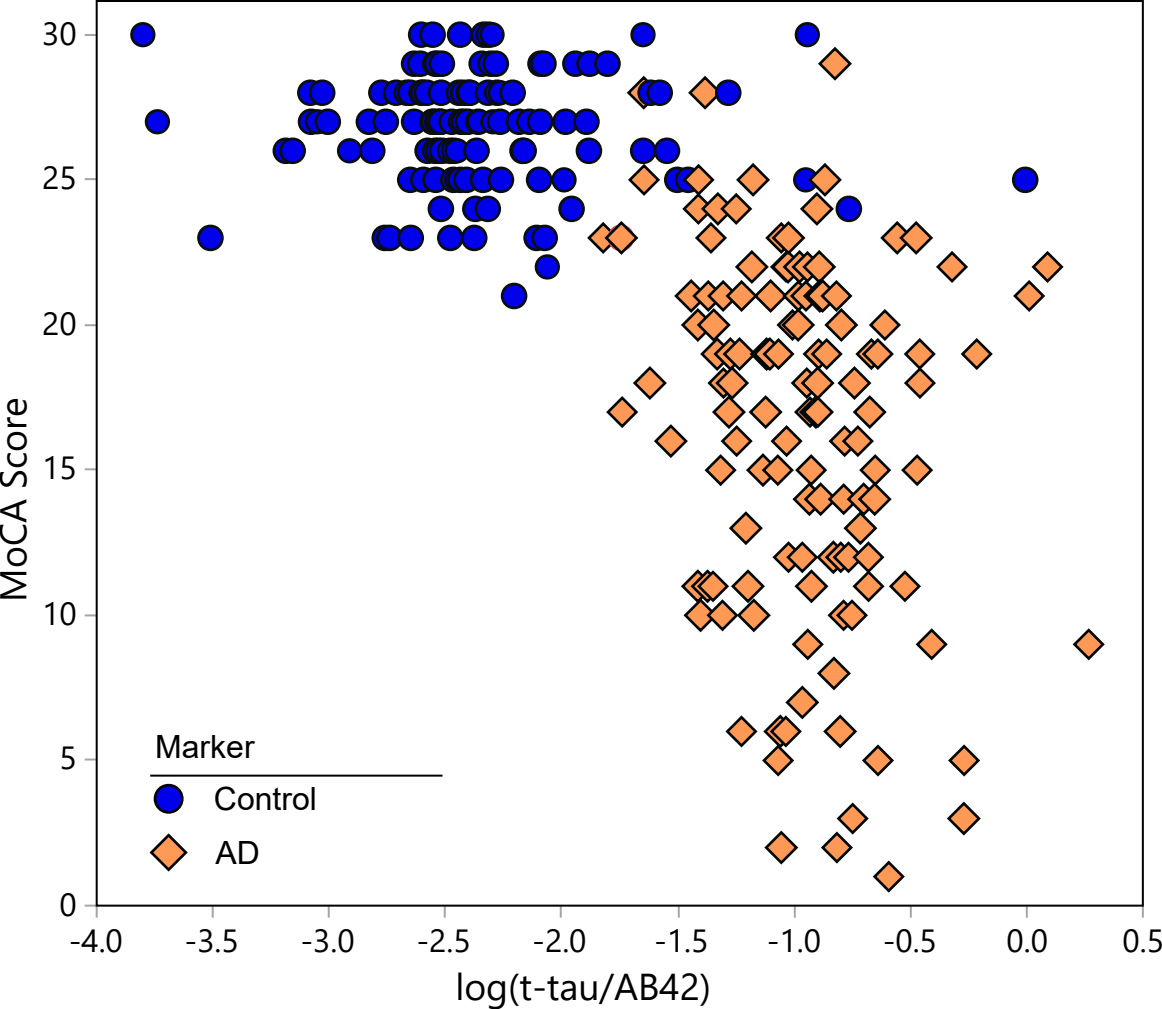

**Figure S3.** Control and AD group MoCA and  $\log(t\text{-Tau}/A\beta_{42})$ .

Supplement: Supplementary file 3 — Additional file 3 : Figure S3. Control and AD group MoCA and log(t-Tau/Aβ42). [file 13195_2021_893_MOESM3_ESM.pdf]
