# Supplementary material for: Association of plasma and CSF cytochrome P450, soluble epoxide hydrolase, and ethanolamide metabolism with Alzheimer’s disease
Source: Alzheimers Res Ther. 2021 Sep 6;13:149. doi: 10.1186/s13195-021-00893-6 (PMC8422756; doi:10.1186/s13195-021-00893-6)
Supplement: Supplementary file 9 — Additional file 9 : Table S6. Spearman's rank order correlation between AD-related markers and plasma lipid mediators. Analysis performed utilizing only subjects predicted to be fasted with the probability >60%, including 60 AD patients and 96 healthy controls. Only associations with the p<0.05 are displayed. Associations colored based on Spearman's ρ (values shown in the table), with blue color for negative and orange for positive associations. [file 13195_2021_893_MOESM9_ESM.pdf]

Table S6. Spearman's rank order correlation between AD-related markers and plasma lipid mediators. Analysis performed utilizing only subjects predicted to be fasted with the probability >60%, including 60 AD patients and 96 healthy controls. Only associations with the $p<0.05$ are displayed. Associations colored based on Spearman's ρ (values shown in the table), with blue color for negative and orange for positive associations.

Variable	log(t-Tau/AB42)	AB42	pTau	pTau/tTau	tTau	MoCA
8-HETE	-0.301	0.191	-0.225		-0.313	
14_15-DiHETrE	0.299	-0.173	0.161	-0.167	0.277	-0.236
17_18_DiHETE+19_20_DiHDoPe	0.31	-0.22		-0.241	0.267	-0.29
17_18-DiHETE	0.38	-0.3	0.169	-0.252	0.318	-0.355
AEA	-0.33	0.302	-0.215		-0.276	0.198
DEA	-0.329	0.247	-0.26		-0.316	0.196
DEA/aLEA	-0.368	0.223	-0.288		-0.383	0.273
DEA/LEA	-0.454	0.322	-0.368		-0.447	0.346
DHEA/aLEA	-0.345	0.237	-0.185	0.168	-0.316	0.313
DHEA/LEA	-0.403	0.349	-0.229		-0.33	0.382
DHEAS	-0.356		-0.353		-0.43	0.253
OEA/LEA	-0.424	0.43	-0.25		-0.311	0.419
DHEA	-0.293	0.259	-0.174		-0.242	0.229
11_12-DiHETrE	0.28	-0.161		-0.174	0.266	-0.215
Progesterone	-0.277	0.199	-0.242		-0.293	0.266
9-HEPE	-0.268	0.295		0.216	-0.199	
(GDCA+GLCA)/(TDCA+TLCA)	0.258	-0.255	0.164		0.192	
12,13-DiHOME/EpOME 2	-0.255	0.179	-0.177		-0.239	0.159
14-HDoHE	-0.255	0.195	-0.208		-0.236	
4-HDoHE	-0.245	0.283				0.178
OEA	-0.243	0.271			-0.164	0.17
9_12_13-TriHOME	-0.239	0.217	-0.165		-0.183	0.162
9_10-DiHODE/9(10)-EpODE	0.237	-0.181	0.219		0.184	
DGLEA	-0.229	0.204	-0.203		-0.204	
F2-IsoP	-0.217	0.171			-0.209	0.21
Sum(HDoHEs)	-0.214	0.192	-0.162		-0.195	
Sum(DiHOME)/Sum(EpOME)	-0.212		-0.183		-0.223	
GLCA	0.21				0.197	-0.178
GDCA/DCA	0.201	-0.16			0.178	-0.177
(TDCA+GDCA)/DCA	0.19				0.165	-0.172
9_10-e-DiHO	-0.188	0.195				
12-HEPE	-0.188	0.196				0.174
AA	0.188		0.177		0.207	
12-HEPE/12-HETE	-0.186	0.198				0.173
GHDCA	-0.184				-0.168	

Spearmans ρ	color
0.1 to 0.2	
0.2 to 0.3	
0.3 to 0.4	
> 0.4	
-0.1 to -0.2	
-0.2 to -0.3	
-0.3 to -0.4	
< -0.4	

Continuation of the Table S7

Variable	log(t-Tau/AB42)	AB42	pTau	pTau/tTau	tTau	MoCA
9(10)-EpOME	0.181				0.215	
5-HEPE	-0.177	0.209				
w+a+(b)-MCA	0.176				0.202	-0.187
Tes/Prog	0.17	-0.171			0.162	-0.161
5-HETE	-0.167	0.178				
DCA/(LCA+UDCA)	-0.167					
15(16)-EpODE	0.165				0.17	
9,10-DiHOME/EpOME 2	-0.164				-0.195	
PGF2a	-0.162		-0.182		-0.175	
8_9-DiHETrE	0.157	-0.161				
NA-Gly					0.199	-0.175
1-OG						
TXB2		0.199				
GDCA/CA						-0.197
CA/CDCA		0.168				0.3
PGE2						
13-HODE		0.193				
GCA/GCDCA						0.179
15_16-DiHODE/15(16)-EpODE						
(w)+a+b-MCA						
GCA/CA		-0.162				-0.165
11,12/14,15-DiHETrE						
14,15/11,12-DiHETrE						
13-KODE						
w-MCA/UDCA					-0.169	
(GCA+TCA)/CA						-0.157
ALA						
12(13)-EpOME						
GDCA						-0.192
TDCA/GDCA						
GCDCA						-0.182
Sum(DiHODE)/Sum(EpODE)						
GCDCA/GLCA						
GDCA/GLCA						
PGD2						0.18
LA						
w-MCA/T-a-MCA						
(TUDAC+GUDCA)/UDCA						
9(10)-EpODE			-0.179			
1-AG						

Continuation of the Table S7

Variable	log(t-Tau/AB42)	AB42	pTau	pTau/tTau	tTau	MoCA
EPA						
11(12)-EpETrE						-0.159
CA						
TDCA/CA						-0.179
2-OG				0.171		
(TDCA+TCDCA)/(GDCA+GCDCA)						
9_10-DiHOME						
Cortexolone						
12_13-DiHOME						
TCDCA/GCDCA						
9_10-DiHODE						
1-LG						
GLCA/CDCA						
13-HOTE						
TCA/CA						
GCA/GDCA						
LCA/CDCA						
9-HETE						
corticosterone						
12-HETE						
EPEA/LEA						
UDCA/CDCA						
T-w+(a)+b-MCA						
TUDCA						
EPEA/aLEA						
Testosterone						
TLCA/CDCA						
LCA						
15_16-DiHODE						
TDCA/TLCA						
UDCA						
9-HODE						
14(15)-EpETrE				-0.171		
11(12)/14(15)-EpETrE						
Cortisone						
11-HETE						
DCA/CA						
TDCA/DCA						

Continuation of the Table S7

Variable	log(t-Tau/AB42)	AB42	pTau	pTau/tTau	tTau	MoCA
GUDCA						-0.159
(GLCA+TLCA)/LCA						
13-KODE/13-HODE						
TCA						
Cortisol						
2-AG				0.17		
T-a-MCA/CDCA						
(TCA+GCA+TDCA+GDCA)/(GUDCA+TUDCA+GLCA+TLCA+TCDCA+GCDCA)						
EPEA						
TCDCA/CDCA						
CDCA						
15-HEPE						
GCA						
12_13-DiHODE						
LEA						
TDCA						
19_20-DiHDoPE				-0.216		
17-OH Prog						
aLEA						
15-HETE						
2-LG						
Sum(DiHETrE/EpETrE)						
5_15-DiHETE						
POEA						
TCDCA						
TLCA						
NO-Gly						
GCDCA/GDCA						
POEA/LEA						
DCA						
DHA						
(GDCA+TDCA)/(TUDCA+GUDCA)						
GCDCA/CDCA						
9-HOTE						
14_15-DiHETrE/14(15)-EpETrE						
5_6-DiHETrE						
(GDCA+TDCA)/(TLCA+GLCA)						
(TCDCA+GCDCA)/CDCA						
