# Supplementary material for: Association of plasma and CSF cytochrome P450, soluble epoxide hydrolase, and ethanolamide metabolism with Alzheimer’s disease
Source: Alzheimers Res Ther. 2021 Sep 6;13:149. doi: 10.1186/s13195-021-00893-6 (PMC8422756; doi:10.1186/s13195-021-00893-6)
Supplement: Supplementary file 10 — Additional file 10 : Table S7. Spearman's rank order correlation between AD-related markers and CSF lipid mediators. Analysis performed utilizing 151 AD patients and 142 healthy controls. Only associations with the p>0.05 are displayed. Associations colored based on Spearman’s ρ (values shown in the table), with blue color for negative and orange for positive associations. [file 13195_2021_893_MOESM10_ESM.pdf]

**Table S7**. Spearman's rank order correlation between AD-related markers and CSF lipid mediators. Analysis performed utilizing 151 AD patients and 142 healthy controls. Only associations with the p>0.05 are displayed. Associations colored based on Spearman's ρ (values shown in the table), with blue color for negative and orange for positive associations.

| Spearmans ρ | color |
|---|---|
| 0.1 to 0.2 | |
| 0.2 to 0.3 | |
| 0.3 to 0.4 | |
| > 0.4 | |
| -0.1 to -0.2 | |
| -0.2 to -0.3 | |
| -0.3 to -0.4 | |
| < -0.4 | |

| **Variable** | **log(t-Tau/AB42)** | **AB42** | **pTau** | **pTau/tTau** | **tTau** | **MoCA** |
|---|---|---|---|---|---|---|
| 9(10)/12(13)-EpOME | -0.232 | 0.195 | -0.13 | 0.147 | -0.218 | 0.176 |
| 9(10)-EpOME | 0.259 | -0.242 | 0.144 | | 0.213 | -0.19 |
| 12(13)-EpOME | 0.303 | -0.285 | 0.169 | | 0.249 | -0.217 |
| AA | -0.263 | 0.258 | -0.186 | | -0.19 | 0.234 |
| EPA | -0.266 | 0.253 | -0.167 | | -0.19 | 0.215 |
| LA | -0.289 | 0.233 | -0.226 | | -0.276 | 0.258 |
| OEA | -0.328 | 0.308 | -0.222 | | -0.283 | 0.329 |
| OEA/LEA | -0.382 | 0.346 | -0.254 | 0.136 | -0.335 | 0.343 |
| Sum(EpOMEs) | 0.283 | -0.268 | 0.155 | | 0.231 | -0.206 |
| Sum(LA Cyp+sEH) | 0.284 | -0.273 | 0.157 | | 0.23 | -0.218 |
| F2-IsoP | 0.2 | | 0.146 | -0.157 | 0.242 | -0.12 |
| 9_10-DiHOME | 0.185 | -0.221 | | | | -0.19 |
| GCDCA/GLCA | -0.176 | 0.161 | | | | 0.174 |
| Sum(DiHOMEs) | 0.169 | -0.2 | | | | -0.189 |
| LEA | 0.166 | | 0.154 | | 0.17 | -0.119 |
| 14_15-DiHETE | -0.162 | 0.2 | | | -0.119 | |
| DHA | -0.157 | 0.193 | | | | 0.151 |
| 11,12/14,15-DiHETrE | -0.153 | | -0.179 | | -0.154 | 0.159 |
| 14,15/11,12-DiHETrE | 0.153 | | 0.179 | | 0.154 | -0.159 |
| 12_13-DiHOME | 0.152 | -0.175 | | | | -0.178 |
| ALA | -0.145 | 0.122 | -0.125 | | -0.123 | 0.154 |
| (GDCA+GLCA)/(TDCA) | 0.143 | | | | 0.137 | -0.163 |
| T-a-MCA | 0.142 | -0.181 | | | | -0.142 |
| GCDCA/GDCA | -0.136 | | | | | |
| TDCA/GDCA | -0.132 | | | | -0.137 | 0.146 |
| GLCA | 0.132 | -0.166 | | | | -0.154 |
| TCDCA/CDCA | -0.126 | | | | -0.138 | 0.177 |
| 12,13-DiHOME/EpOME | -0.124 | | | | -0.12 | |
| PGF2a | -0.123 | 0.16 | | | | |
| 11-Deoxy-CTRL | 0.121 | -0.12 | 0.119 | | | -0.135 |
| DCA | 0.121 | | | | | -0.13 |
| UDCA | 0.12 | | | -0.167 | 0.16 | |
| GCA/GCDCA | 0.117 | | | | | |
| CRTL | | | | -0.185 | | |
| (TDCA+TCDCA)/(GDCA+GCDCA) | | | | | -0.127 | 0.123 |

Continuation of the Table S7

| **Variable** | **log(t-Tau/AB42)** | **AB42** | **pTau** | **pTau/tTau** | **tTau** | **MoCA** |
|---|---|---|---|---|---|---|
| UDCA/CDCA | | | | -0.162 | 0.144 | |
| CRTN | | | | | | -0.151 |
| TCDCA/GCDCA | | | | | -0.136 | 0.119 |
| 13-HOTE | | | | | | |
| 20-HETE | | | | | | |
| (TCDCA+GCDCA)/CDCA | | | | | -0.115 | 0.165 |
| TCDCA | | | | | -0.118 | |
| GDCA | | | | | | |
| GCDCA/CDCA | | | | | | 0.161 |
| 14,15/17,18-DiHETE | | 0.135 | | | | |
| EPA + DHA diols | | | | | | |
| w-MCA | | | | | | |
| TCA | | | | | | |
| 11_12-DiHETrE | | | | | | |
| TDCA/DCA | | | | | | 0.124 |
| TDCA/DCA 2 | | | | | | 0.124 |
| T-a-MCA/CDCA | | | | | | |
| (TUDAC+GUDCA)/UDCA | | | | | | |
| GUDCA/UDCA | | | | | | |
| GLCA/CDCA | | | | | | |
| Sum(DiHOME)/Sum(EpOME) | | | | | | |
| 15_16-DiHODE | | | | | | |
| TEST | | | | | | -0.148 |
| 17_18-DiHETE | | | | | | |
| 9,10/12,13-DiHOME | | | | | | |
| TUDCA/UDCA | | | | | | |
| (GDCA+TDCA)/(TLCA+GLCA) | | | | | | |
| GCA/GDCA | | | | | | |
| TUDCA | | | | | | |
| GUDCA | | | | -0.125 | | |
| CRCTN | | | | | | |
| 9-HOTE | | | | | | |
| DCA/(LCA+UDCA) | | | | 0.126 | | |
| GCDCA | | | | | | |
| CDCA | | | | | | |
| GDCA/GLCA | | | | | | |
| GCA | | | | | | |
| (TDCA+GDCA)/DCA | | | | | | |
| DHEA/LEA | | | | -0.144 | | |

Continuation of the Table S7

| **Variable** | **log(t-Tau/AB42)** | **AB42** | **pTau** | **pTau/tTau** | **tTau** | **MoCA** |
|---|---|---|---|---|---|---|
| w-MCA/UDCA | | | | | | |
| 19_20-DiHDoPE | | | | | | |
| GDCA/DCA | | | | | | |
| 17OH-PROG | | | | | | |
| a-MCA | | | | | | |
| 13-HODE | | | | | | |
| 9-HODE | | | | | | |
| 14_15-DiHETrE | | | | | | |
| (GDCA+TDCA)/(TUDCA+GUDCA) | | | | | | |
| 9,10-DiHOME/EpOME | | | | | | |
| DHEA | | | | -0.163 | | |
| TDCA | | | | | | |
| w-MCA/T-a-MCA | | | | | | |
