# Supplementary material for: Association of plasma and CSF cytochrome P450, soluble epoxide hydrolase, and ethanolamide metabolism with Alzheimer’s disease
Source: Alzheimers Res Ther. 2021 Sep 6;13:149. doi: 10.1186/s13195-021-00893-6 (PMC8422756; doi:10.1186/s13195-021-00893-6)
Supplement: Supplementary file 11 — Additional file 11 : Table S8. Stepwise logistic model predicting AD status using plasma, CSF or both plasma and CSF metabolites. Stepwise analysis was performed with the maximal validation r2 as the model stopping criteria, or if an additional step increased the BIC. Model stopping point for each analysis is highlighted. [file 13195_2021_893_MOESM11_ESM.pdf]

Table S8. Stepwise logistic model predicting AD status using plasma, CSF or both plasma and CSF metabolites. Stepwise analysis was performed with the maximal validation r^2 as the model stopping criteria, or if an additional step increased the BIC. Model stopping point for each analysis is highlighted.

Step	Parameter	L-R ChiSquare	Sig Prob	Entry ChiSquare	Entry "Sig Prob"	RSquare	AICc	BIC	RSquare Validation
				**Plasma**					
1	DEA/LEA	32	0.0001	27.8	1.36E-07	0.219	118	124	0.153
2	OEA/LEA	16.7	0.0001	15.1	1.03E-04	0.333	104	112	0.425
3	12,13-DiHOME/EpOME	11.5	0.0007	10.5	1.21E-03	0.412	94.4	105	0.469
4	14_15-DiHETrE	11.5	0.0007	11.3	7.95E-04	0.491	85.1	97.9	0.512
5	9_12_13-TriHOME	7.07	0.0078	6.46	0.0111	0.539	80.2	95.6	0.559
6	**CA/CDCA**	**4.87**	**0.0274**	**4.54**	**0.0331**	**0.572**	**77.7**	**95.4**	0.62
7	13-KODE	3.03	0.0817	3.02	0.0824	0.593	77	97.1	0.65
				**CSF**					
1	CSF_OEA/LEA	43.7	0.0001	39.1	3.96E-10	0.154	244	251	0.0935
2	CSF_12(13)-EpOME	25.8	0.0001	24	9.75E-07	0.245	221	231	0.13
3	CSF_9(10)-EpOME	9.26	0.0023	8.86	0.00292	0.277	213	227	0.152
4	**CSF_GLCA**	**7.46**	**0.0063**	**7.33**	**0.00677**	**0.304**	**208**	**224**	0.167
5	CSF_12_13-DiHOME	3.72	0.0537	3.6	0.0577	0.317	207	226	0.183
				**CSF and plasma**					
1	DEA/LEA	32	0.0001	27.8	1.36E-07	0.219	118	124	0.153
2	CSF_OEA/LEA	17.4	0.0001	16.7	4.36E-05	0.337	103	111	0.18
3	DHEA/LEA	8.11	0.0044	7.5	6.19E-03	0.393	97.2	108	0.284
4	17_18-DiHETE	6.35	0.0117	6.29	1.22E-02	0.436	93	106	0.384
5	**9_12_13-TriHOME**	**6.14**	**0.0132**	**5.77**	**0.0163**	**0.478**	**89.1**	**104**	0.42
6	CA/CDCA	1.54	0.214	1.53	0.216	0.489	89.9	108	0.473
